# Why things look the way they do: we live in a Cartesian grid of reified concepts

**DOI:** 10.3389/fpsyg.2025.1612191

**Published:** 2025-09-11

**Authors:** Guy C. Brown

**Affiliations:** Cambridge Neuroscience, University of Cambridge, Cambridge, United Kingdom

**Keywords:** reified concepts, extended mind, distributed cognition, architecture, aesthetics, philosophy, phenomenology, environmental psychology

## Abstract

The world we live in now is almost exclusively populated by things designed by minds to be understood by other minds. I argue here that: (1) Human environments now consist mainly of reified concepts, such as “chairs.” (2) These externalized concepts look like simple cartoons of the concepts they reify, with flat, homogenous surfaces in geometric shapes, with one or a small number of colors, textures and surfaces, so that they can be easily identified and distinguished. (3) Reified concepts are organized within a Cartesian grid, that enables their perception, location and memory. (4) Simple concepts are nested within complex concepts, such as rooms, houses, streets, hospitals or cities, designed to be read by our minds, and guide behavior, within the externalized mind of society. (5) Components of the human environment that are not conceptual are actively removed, resulting in a very low entropy of information, and giving the illusion that reality is entirely conceptual. (6) Reified concepts and their conceptual ordering help perception, comprehension and use of human environments. (7) These ideas have application to design, architecture, aesthetics, phenomenology, ontology and understanding why things look the way they do.

## 1 Introduction

Why do things look the way that they do? This is an ambiguous question, that can be understood at different levels, but the question is fundamental. A variety of approaches have been used to address this or related questions. Philosophers have theorized the relation between perception and the world (Locke, 1690; Kant, 2007). Gestalt psychologists and phenomenologists have explored the rules governing the phenomenology of perception (Koffka, 1922; Merleau-Ponty, 1945; Albright, 1994). Semiotic approaches have interpreted the human environment in terms of signs and what is signified by the creator and/or the interpreter of the sign (Maran, 2007; Gottdiener and Lagopoulos, 1986). Environmental psychology and cognitive geography have examined how humans relate to the spaces around them (Gibson, 1979; Norton, 1997). The relation between aesthetics, cognitive processing and the simplicity of the environment has been investigated for nearly a century (Birkhoff, 1933; Reber et al., 2004). Evolutionary human psychology has explained how we see the non-human environment in terms of past selection pressures on perception and survival (Shimura and Palmer, 2012; Gaulin and McBurney, 2003). Cognitive neuroscience and psychology has unraveled the mechanisms of perception and how they shape how thing look (Barlow, 1982; Gilbert and Wiesel, 1990).

However, I am interested here in why the world around us looks the way it does, from a different perspective to those cited above. I think that how things look in modern human environments, reveals something surprising about the composition of those environments, i.e., that they are almost exclusively composed of reified concepts embedded within conceptual structures, designed to help our minds see, locate and remember those concepts. And this understanding of the nature of human environments can help explain why such environments look the way they do. We normally think of concepts as passive descriptors of the world, that evolve to fit that world better. But in this scenario, concepts actively shape the external world, and are then used to read that world, resulting in a positive feedback loop, by which the external world becomes more like the concepts, until eventually we are living in a world of reified concepts. (By “reified concepts” or “externalized concepts”, I mean concepts that have been made into objects, that are partly designed to invoke the concept in our minds).

The ideas outlined here relate to a variety of previous ideas of others. In 1781, Immanuel Kant proposed that the world, as we experience it, is constructed by the mind, so that space, time, objects and causation are ways in which that experience is structured by our minds (Kant, 2007). In 1950s and 60s, Roland Barthes and Jean Baudrillard extended semiotics to the human environment, interpreting it in terms of signs and connotations (Barthes, 1997; Baudrillard, 2005). In 1976, the evolutionary biologists, Richard Dawkins proposed “memes” as units of cultural inheritance that spread from mind to mind (Dawkins, 1976); and in 1982, Dawkins used the idea of the “extended phenotype” to refer to things or structures made by people or other animals (such as a spider's webs) to aid their fitness (Dawkins, 1982). In 1995, the anthropologist Edwin Hutchins introduced the idea of “distributed cognition”: that the cognitive acts of people are distributed across multiple people, tools and the human environment, rather than being confined to an individual's mind (Hutchins, 1995). In 1998, the philosophers Andy Clark and David Chalmers proposed that some objects in the external environment can be part of a cognitive process and thereby function as extensions of the mind, and they called this idea: “the extended mind” (Clark and Chalmers, 1998; Wilson, 2002). All of these ideas are complimentary but different to the idea proposed here (that the human environment now consist of reified concepts), and are of relatively little help in understanding why human environments look the way that they do.

## 2 How we see

In order to understand why the human environment looks the way it does, we need to understand how we see and model the world. We naively think of “seeing” as a direct experience, or passive witnessing, of the world, as envisioned by direct/naïve realism. But we now know (from experiments and lesions on human and animal brains) that the world is perceived actively and indirectly via collecting intermittent and fragmentary sensory data that is analyzed to update a best-guess model of the world (Gregory, 1966; Stone, 2012; Seth, 2021). There are two types of model used by our minds in visual perception: (i) pictorial, e.g., a distribution color in the visual field, as in a picture, and (ii) linguistic/conceptual, e.g., “the cat is sat on the mat” as a description/model of the scene in words/concepts. These roughly correspond to the dorsal and ventral streams of visual processing, ending in the parietal and temporal lobes of the brain, respectively (Mishkin and Ungerleider, 1982; Goodale and Milner, 1992; Norman, 2002; Graumann et al., 2022).

Analogously, we may think of the world that we are seeing as containing: (i) a distribution of matter in the space of the external world, and/or (ii) a set of concepts or facts about the world (Locke, 1690; Wittgenstein, 1922). However, the current scientific conception of the world and perception (indirect realism) considers that concepts are confined to minds, and are not found in the world outside us. I argue here that human environments are now designed, constructed and curated to be easily read at the linguistic/conceptual level, rather than perception stalling at the pictorial level due to slow, ambiguous or wrong understanding of what is seen (Norman, 1999).

We do not “see” the whole visual scene, but rather we scan it for interesting things, using tunnel vision, visual analysis and prediction (Stone, 2012; Seth, 2021). For example, only central vision in the fovea can make fine visual distinctions, so the eyes rotate (saccade) two or three times a second to sample the visual field, hunting for interesting visual input. And that interesting visual input has to be found by processing, for example, neurons in V1 of the visual cortex are connected up to detect edges with specific orientations in the visual input. And subsequent neurons in the visual stream detect specific shapes in the scene. And these neurons may activate neuronal networks in the temporal cortex trained to detect specific objects, nouns or concepts (Stone, 2012). But specific objects in the environment can stimulate multiple neuronal networks, which may also be stimulated by non-visual stimuli, so such neuronal networks are predicting the visual stimulus based on prior concepts and a variety of information, rather than simply detecting the stimulus. In the parietal cortex, the location (rather than the conceptual identity) of the stimulus is predicted. If the predicted stimulus is sufficiently interesting, it may induce conscious thinking about the stimulus, but conscious thinking occurs one thought at a time, and is slow, so has limited capacity to analyze visual input (Gregory, 1966; Seth, 2021).

The important point here is that the visual scene is not passively witnessed, but rather has to be actively analyzed and predicted, using limited information and resources, resulting in uncertain perception, based on prior concepts. Thus, things that do not signify what they are, or correspond to what we are expecting, may not be seen at all. Consequently, it is necessary to make the human environment as simple as possible to understand, given the ways in which our minds extract content and location from the visual scene.

Human environments may also be optimized for memory, and memory is important because only things that are remembered survive in our minds. Psychology experiments show that visual memory for objects that are meaningful and conceptually distinct is very high, whereas memory for objects or features that are meaningless is very low (Konkle et al., 2010; Shoval et al., 2022). Features of the environment that can be named are better remembered; similarly, features that are orientated to the vertical or horizontal (i.e., to the grid) are better remembered (Pereira Seabra et al., 2025). Therefore, the human environment may be designed to be processed through to the linguistic/conceptual level, partly in order to be remembered.

## 3 Scenes from an interior life

In this article, I want to point out something fundamental to our modern world, but this can seem either very obvious or very obscure, depending on the reader's background. So, I need to steer the reader between these twin perils, in order to see the world anew from this perspective. In this section, I am going to start, rather informally, by looking at some photographs of the environments around me as I write this article. Note that this article is written from the perspective of a white male in a western culture, and the extent to which the conclusion generalize to other people and cultures, would require further investigation.

**Figure 1:**
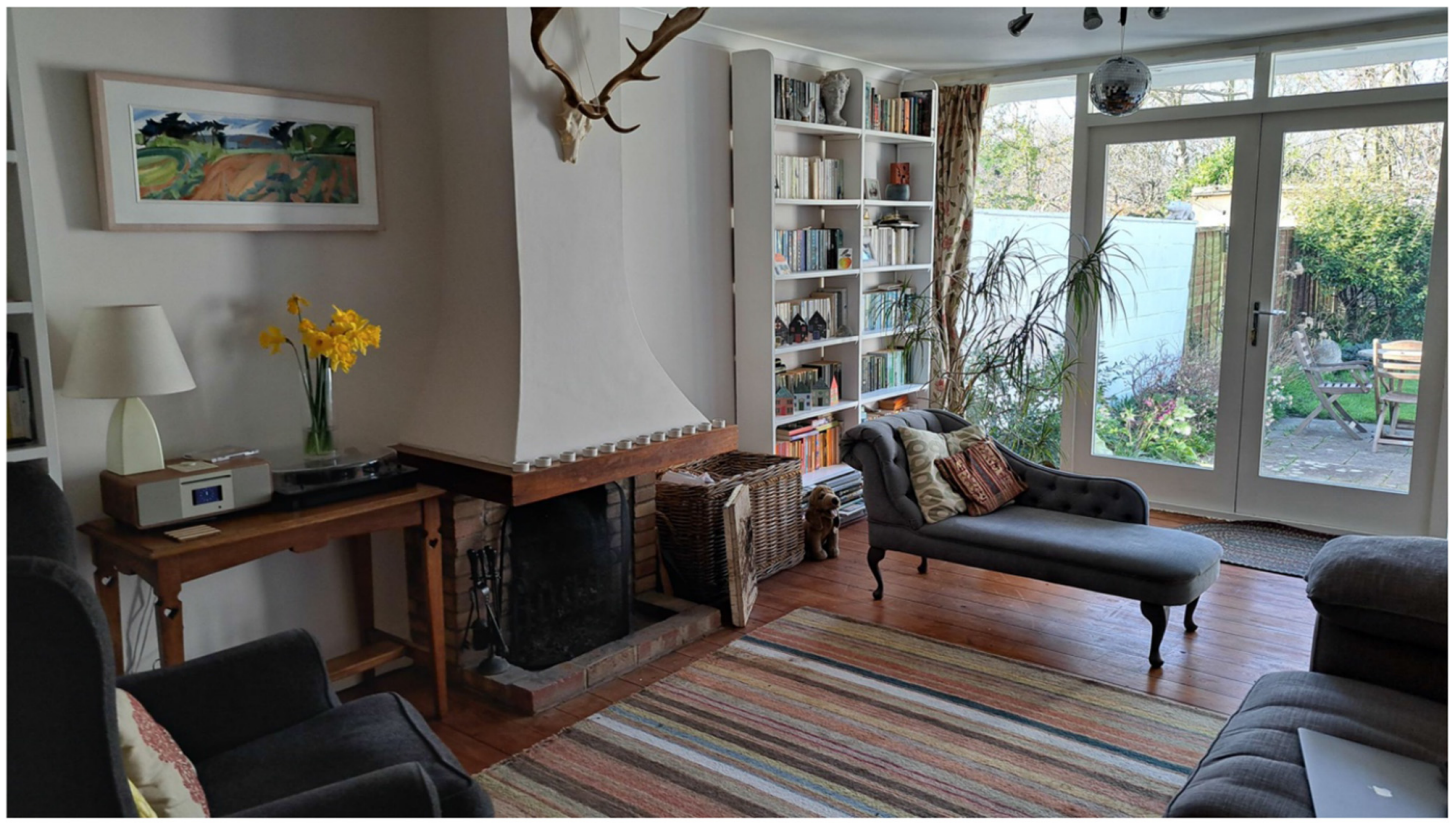
A domestic scene, consisting almost entirely of reified concepts orientated to a Cartesian grid.

Let's look at this photograph (Figure 1), representative of a domestic scene. It happens to be the scene in front of me now, as I write this, but that's not important: I have not rearranged this scene. There are a variety of things arranged in this space, and it looks pretty boring and uninteresting, but that's because we understand the scene and almost everything in it. We understand the scene because we can “read” the objects and their arrangement within it. And we can do that because there are only unambiguous objects in well-known arrangements, and almost nothing else.

The scene contains objects, with names, such as chairs, table, carpet, lights, books, fireplace, walls, floor, ceiling, doors, garden, and room. Almost everything can be named—although there is one semi-ambiguous object near the center of the photograph, for contrast. The objects can be named, because they are designed to evoke the name in our minds by looking like a cartoon of the name. For example, the chairs and table, have four vertical legs, supporting a horizontal surface, and the chairs have a back on top. Each object generally has the same color over all of its parts, in order for it to pop-out as an object in our minds, in contrast to the background. The objects generally are of very-low entropy, and are geometrically shaped (or have geometrically-shaped parts) with flat, homogenous surfaces.

And the arrangement of objects or parts is generally geometric. For example, the room is an oblong box shape, with flat, homogenous walls, ceiling and floor, arranged horizontally or vertically, at right-angles to each other. The surfaces are generally flat and homogenous, so that you can see nothing but the conceptual object. For example, the walls are flat and featureless, so that you can see nothing but “walls”—not even the substance of the walls. Straight lines are everywhere, and the room, and almost everything in it, are arranged on a Cartesian grid.

A Cartesian grid is a set of three axes at right angles to each other in three-dimensional space. The axes in this scene are set by the box shape of the room, but these axes may align with the axes of the house, the street or even the city. All straight lines, planes and objects in this scene are aligned to one or more of these three axes—most objects are aligned to all three axes. And the lines in the image enable the viewer to locate all objects within the grid, i.e., the lines function as Cartesian grid lines.

From a physics and information perspective, the scene is very low entropy, i.e., it is highly ordered, and very far from a random distribution of matter, color or pixels. Low entropy structures and arrangements are unnatural, according to the second law of thermodynamics, and require considerable effort to create and maintain. So why is this scene and its objects (and the human environment generally) so low entropy? I will argue that the human environment is low entropy, and thus highly ordered, because it was made by minds, and the low entropy aids comprehension of the scene. There is extensive evidence that when looking at a scene, people attend to areas with high visual or semantic information and avoid areas of the scene with low information or low meaning (Loftus and Mackworth, 1978; Henderson and Hollingworth, 1999; Henderson et al., 2019; Henderson and Hayes, 2017). Thus, by making the background and overall scene low entropy, viewers can concentrate their attention on the intended information content of the scene, i.e., the concepts.

Almost everything in the scene was designed (or arranged) by minds, and designed to be read by our minds. Each object started as a concept (normally a word) in someone's mind, and it is designed to evoke a concept (normally a word) in my mind. For example, a chair starts out as a concept in the designer's mind, which then is used to shape matter into the real chair. But part of the design criteria of a chair is that it evokes the concept of chair in someone who sees it. The object must signify unambiguously what it is in order for it to be “seen” and to be of use to us (Henderson and Hollingworth, 1999). Similarly, part of the design criteria of a “room” is that it looks like a room, and nothing else. When I look at a room, I can “read” it, because its parts and all the objects in it are made of words. Each object in the room functions like a word on the page. The Cartesian alignment of all objects in the room may make it easier to locate, read and remember those words. The blank walls, ceiling and floor function in part like blank sheets of paper on which words are printed, so that the words or objects are easily discriminated and seen.

The requirement that things must symbolize what they are, constrains the form of those things. An example of something in this scene that is constrained in form by its name is “ceiling.” Generally, ceilings are almost perfectly featureless, flat, horizontal, rectangular, motionless, and one color, so that they are invisible except for the word “ceiling” that they evoke. Evoking “ceiling” is part of evoking “room,” as the flat, featureless ceiling is co-designed with the flat, featureless floor and walls, all to evoke “room.” And “rooms” are designed to be part of evoking and being a “house,” i.e., the flat, featureless interior of the house is co-designed with the flat, featureless exterior of the house to evoke the concept of “house,” which enables us to recognize it as such and know how to use it.

Why are almost all ceilings, walls and floors featureless, flat, horizontal, rectangular, motionless and one color? I suggest three interrelated reasons: (a) because this enables us to read them unambiguously as a “ceiling,” “wall,” “floor” or “room,” (b) because this presents a blank canvas/page on which the objects of the room can be seen relatively unambiguously, and (c) the blank box structure of the room creates the Cartesian grid within which the objects of the room can be located within the mind of the viewer. Conventional explanations are: (i) it is easier and cheaper to build it that way, (ii) it is more functional that way, or (iii) it is more aesthetic that way. To a certain extent it is true that it is easier and cheaper to build a ceiling that is flat and featureless. But the opposite is even more true, i.e., it would be easier and cheaper: to not have a ceiling at all, to not plaster the ceiling or to not skim it perfectly flat, to not paint it at all or to not paint it homogenously. If it was easier and cheaper to build a ceiling that is flat and featureless, we might expect to find flat and featureless ceilings more in poor houses than in wealthy houses, but the opposite is generally the case: poor rural houses may have less flat and featureless ceilings, or no ceiling at all, precisely because it is more expensive to have perfectly flat and featureless ceilings.

Are flat and featureless ceilings more functional? It is hard to see how this could be the case, since the only function ceilings have is to cover the joists, wires and underside of the floor above, and to insulate sound and heat—and flat and featureless ceilings do not do this function better than lumpy and feature-filled ceilings.

Are flat and featureless ceilings more aesthetic? Possibly, but if so, this begs the question why, because a flat and featureless ceiling appears to have no aesthetic features, indeed it appears to be nothing but the word “ceiling.” If this is beautiful, it must be in a very abstract way—we will discuss this in the section on aesthetics below, but for the moment we conclude that a flat and featureless ceiling does not correspond to our normal concept of beautiful. Indeed, in the rare cases that we refer to a beautiful ceiling, we are referring to a ceiling that is not flat and featureless, e.g., that of Kings College Chapel in Cambridge or one with art on it. Is a flat and featureless ceiling more restful? Possibly, but if so, again, this begs the question why.

As indicated above, I would suggest that ceilings, floors and walls are flat and featureless because: (a) this makes it easier to see ceilings, floors, walls and rooms as the corresponding words or concepts, rather than the actual substance/matter or something ambiguous, (b) it makes it easier to see other conceptual objects against these surfaces, and it lowers the overall entropy of the scene, making it easier to perceive and remember these objects, and (iii) it provides a Cartesian frame for the room, that makes it easier to see the spatial relations between things in the room. I discussed the evidence for these interpretations in the “How we see” section. I have been talking about flat and featureless ceilings, floors and walls, but much the same applies to the flat and featureless surfaces of things within the room and just about everything else designed by minds.

Figure 1 above contains a sub-theme of framed pictures: there is an actual framed picture on the wall, the garden is framed by the doors and windows, the fire would be framed by the fireplace (if it were alight), the books are framed by the bookcases, and the photograph itself is framed by its rectangular outline. In a more abstract way, the walls and room itself, act as a frame for the things displayed against the wall, and within the room. The framing helps demarcate fields within the scene, i.e., the framing helps comprehension of the scene by indicating areas of the scene that need to be understood independently. For example, if the picture on the wall did not have a frame or rectangular outline, then it would be difficult to distinguish from the wall, which would make both the picture and the wall ambiguous. Thus, the framing may instruct the viewer to conceptually treat what is within the frame differently than what's outside the frame.

Almost everything in the scene is orientated on a Cartesian grid: the room is a rectangular box, and all lines of the floor, carpet, furniture, and objects are orientated in conformity with that box. Presumably, this arrangement was unconsciously thought to be tidy, or ordered, or aesthetic. But from the perspective of this article, the Cartesian arrangement of the objects is part of the conceptual structuring of the environment, and what is judged as tidy or aesthetic may be an arrangement that is easy to see, understand and remember.

Why is the human environment orientated to a Cartesian grid? There are a variety of possible explanations. Flat, homogenous surfaces in the background, orientated to the vertical or horizontal axes, help the viewer orientate themselves and the scene, and help judge distance, and thus the layout of the scene, plus the size, and shape of objects (Gibson, 1979; Sinai et al., 1998; Sedgwick, 2021). It is also known that human vision is more sensitive to contours with a horizontal or vertical orientation, compared with oblique orientations, and brain areas involved in large-scale analysis of visual scenes are preferentially activated by such contours or by rectilinear structures, i.e., structures with parallel lines and right angles (Nasr and Tootell, 2012; Nasr et al., 2014). Features of the environment orientated to the horizontal or vertical are also better remembered (Bae, 2021; Pereira Seabra et al., 2025). So, orientation to the Cartesian grid may be preferentially recognized, processed and remembered.

**Figure 2:**
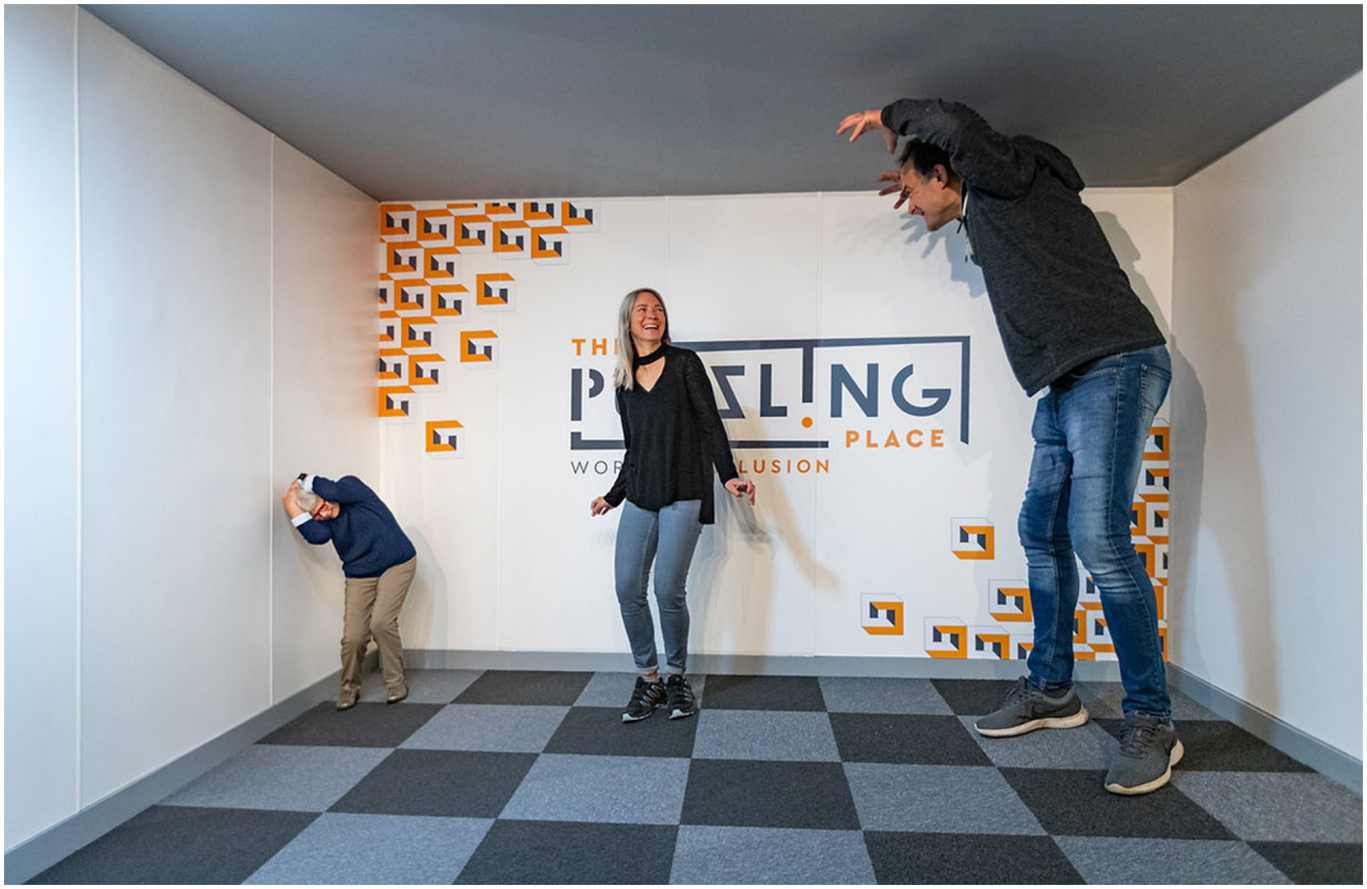
In the Ames room illusion, the Cartesian grid has been systematically distorted to a non-Cartesian grid that distorts our perception of depth, which in turn distorts our perception of relative size, shape and location. The illusion illustrates how important the undistorted Cartesian grid of a room (or street) is in helping us judge the relative depth, shape and size of objects within the grid. Image reproduced with permission from The Puzzling Place.

Also, the relative location of objects in the room (and their size and shape) is not seen directly, it has to be calculated by my brain using a three-dimensional model from two-dimensional sensory data, plus an estimate of depth. It's a pretty complicated calculation, and there are a lot of objects in the room. The Cartesian orientation of objects in the room, makes it easier to understand the relative location and size of objects, which makes it easier to interact with those objects. Linear perspective is an important aid to judging depth in a scene, and therefore estimating the relative size and location of objects, as indicated by the Ames room illusion (Figure 2; Gregory, 1966). Perceiving the relative location of objects within a Cartesian system may also make it easier to remember this information (Pereira Seabra et al., 2025)—for example, remembering the location of a particular book on the bookshelves.

## 4 Alternative scenes

**Figure 3:**
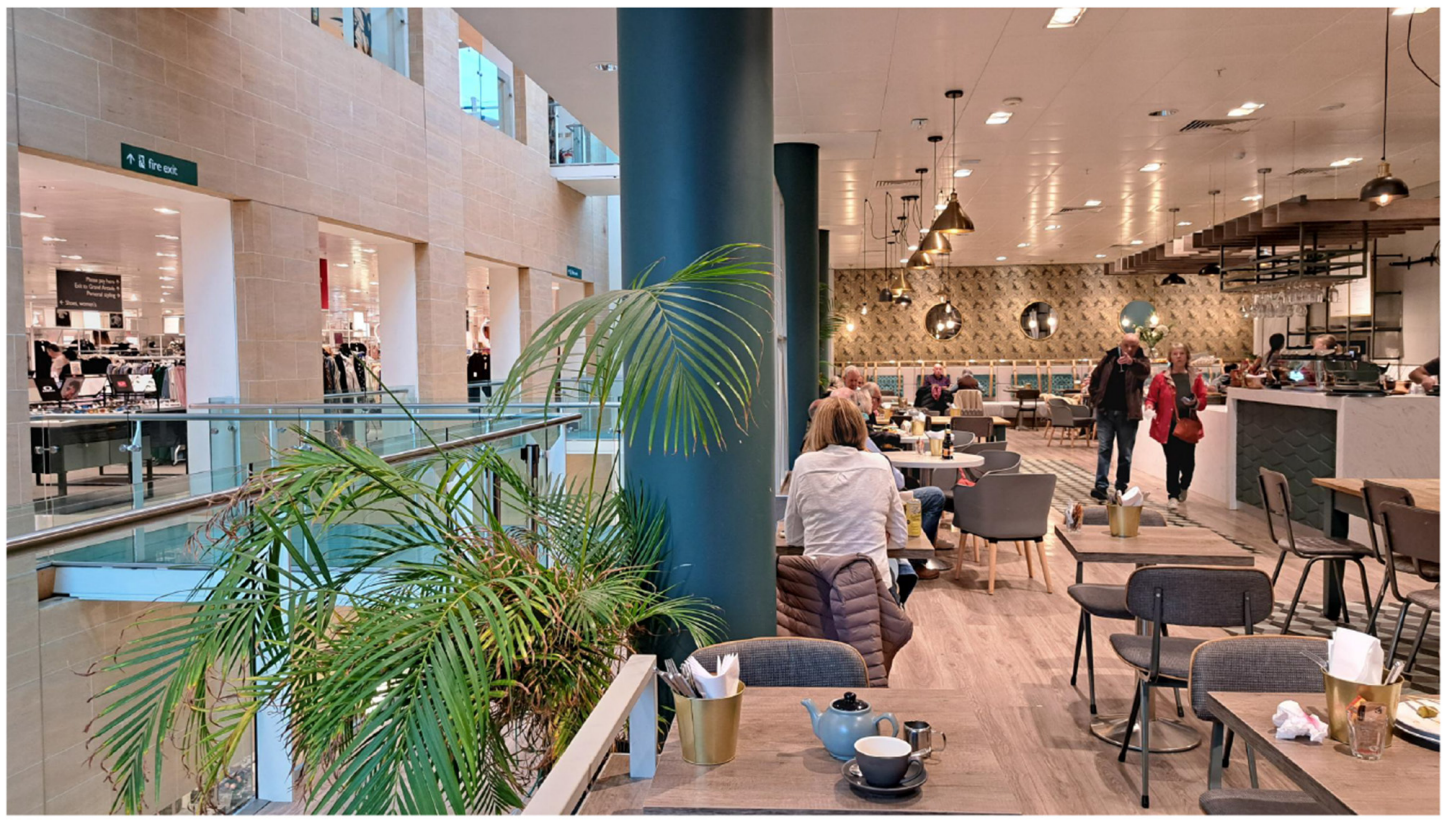
A café in a department store. Notice that most things in the scene are reified objects orientated to a Cartesian grid, which helps to estimate the depth and size of objects.

I have moved location now, and am having a cup of tea in the café of a department store, and I have taken a photograph of the scene in front of me (Figure 3).

This Figure 3 consists mostly of reified concepts, and most such objects are arranged as nested structures in larger concepts, for example, the walls, floor, ceiling and all contents of the café itself. There are repeating units of near identical objects, for example, the chairs, tables, lights, pillars and windows. Almost all surfaces are flat, featureless, and homogenous; and almost all objects are geometric and orientated within a Cartesian grid. The plant is an interesting exception in that the plant and its parts are not orientated to the Cartesian grid set by the room. There is some conceptual framing: of the store on the left, of the café counter on the right, of the café itself by the walls, floor and ceiling, and of what is on the table top by the table top. Colors are used to distinguish objects in the scene: an exception is the floor and table top at bottom of photograph, making it difficult to distinguish these objects. Almost all objects can unambiguously be identified with names. There is minimal non-conceptual content. The entropy of the scene is very low. The Cartesian grid makes it easy to “see” up-down, left-right, and depth axes and distances, and therefore to identify the relative location, size, and shape of objects within the scene, despite the photograph being two dimensional and scene having significant depth.

**Figure 4:**
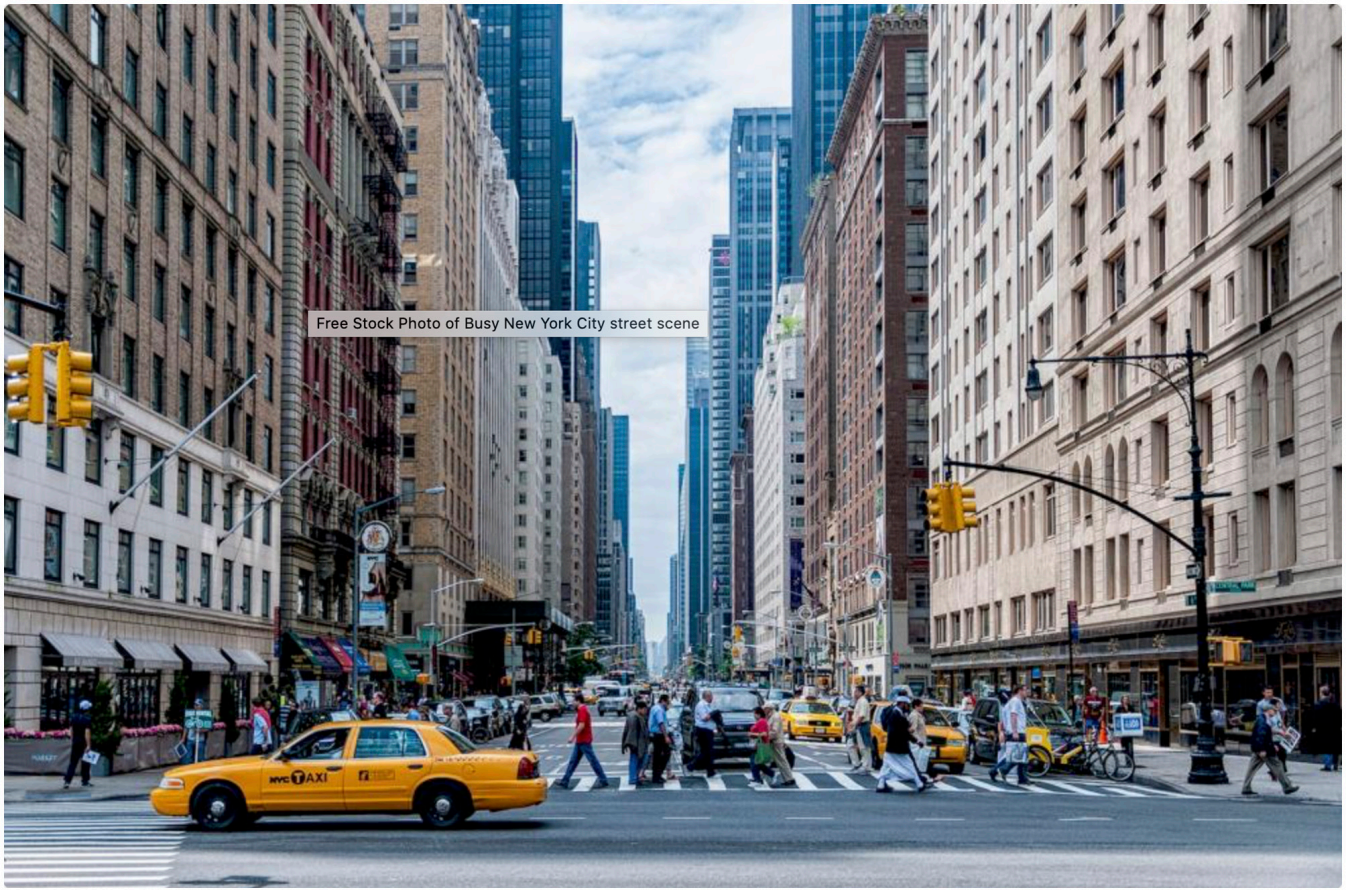
A city street scene, consisting almost entirely of reified concepts, orientated to a Cartesian grid. Photo credit: Frank Kohntopp. CC0 license.

Below, I provide a photograph of an outside human environment (Figure 4).

As with the other human scenes, the image is low entropy, and concept rich. There are lots of identifiable and nameable things present, and relatively little ambiguity about what the things are. One of these (the taxi) even has its name printed on the side. There are lots of horizontal and vertical lines, and almost everything is orientated to a Cartesian grid, which gives a strong sense of up-down and depth, making it relatively easy to estimate the relative size and location of things. The buildings frame the street, making it easier to “see” the street, and judge distance along the street. The surfaces of the road, sidewalks and buildings are relatively homogenous, so that the concepts are seen, rather than the actual matter.

There is a theme of repetition of near-identical units: of taxis, cars, crossing lines, people, windows, and buildings—potentially making it easier to apply the general concept (e.g., window) to the individual thing seen, and requiring less cognitive processing (than if every window were different). The low concept ambiguity, as well as the low location ambiguity, enables pedestrians to confine themselves to the sidewalks and crosswalks, whereas cars confine themselves to the roadways, regulated by traffic lights.

There is also a potential theme of nested concepts. The concepts of Figures 1, 2 are nested within the concept of a room, which is nested within the concept of a building. In Figure 4, a set of windows within a frontage, nests within the concept of a building or building frontage. And a set of these makes up a city block. Or if you combine these with a road and sidewalks etc., you get the concept of a street. And if you combine a set of streets with some other things, you get the concept of a city. Thus, reified concepts overlap, and can be combined into complex conceptual structures.

Complex conceptual structures such as a store or hospital also start out as concepts in someone's mind, and are designed to be seen as a store or hospital, so that the viewer knows what to expect and what to do. The individual conceptual components of the store or hospital (such as a door or counter) guide people through the complex conceptual structures. Businesses, like McDonalds, are designed to be easily understood by our minds: to stand out and be seen, to have a standard form in layout and experience. And within these structures, people can also function as components, but in order to do so, need to be identifiable as, for example, a shop assistant or a nurse. Thus, people can to some extent act as reified concepts.

The externalized concept of a street, depicted in this scene, guides the behavior of both pedestrians, and car drivers, so that they know what to do within the street and in response to different components of the street. The unambiguous clarity of the components of the street is particularly important, when pedestrians and car drivers are moving rapidly through this environment in close proximity. Similarly, the clear Cartesian grid may help estimate the relative location and motion of objects, which is important when collisions between objects could be fatal.

Most reified concepts can be thought of as simple nouns, that describe static entities e.g., a chair or a house. But some reified concepts involve complex, dynamic events over time, e.g., a play, a holiday or going shopping. Again, these things start in someone's mind, and the reified concept is designed to invoke the concept in the participants. Some concepts correspond to verbs, rather than nouns, e.g., driving, shopping or parking, and if the concept is missing from someone, then they will not be able to correctly perceive or understand a scene, such as Figure 4.

Concepts are normally thought of as relatively passive descriptors of a scene, that function entirely within our minds, but here I am suggesting that concepts have a much more active role in constructing human environments, which are almost entirely made up of reified concepts, designed to invoke concepts in the viewer or user of the environment.

## 5 Non-conceptual entities

**Figure 5:**
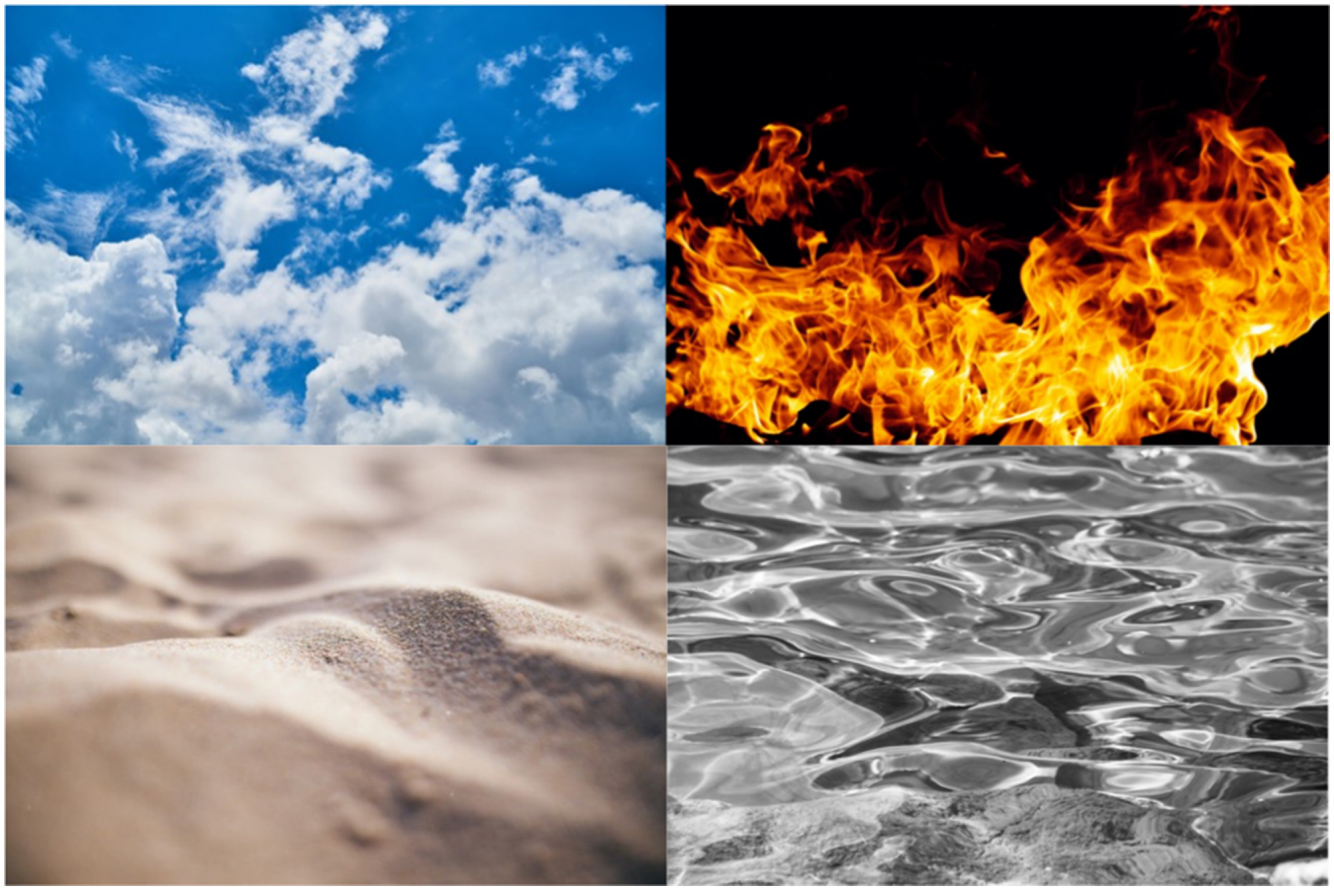
Inanimate, non-human scenes, lacking reified concepts, Cartesian grids or clues to scale, depth or orientation. Photo credits, top left (air): Pixabay; top right (fire): 2happy, bottom left (earth): negativespace.co; and bottom right (water): Anna Romanova. All images: CC0 licenses.

The key point of the above is that: human environments mainly consist of reified concepts, and these are conceptually structured. For contrast with the above human environments, I provide some non-human, inanimate scenes below, which have relatively high entropy and low concept density (Figure 5). It is hard to see anything in the scenes, and therefore hard to remember anything. There is no Cartesian grid, and the images lack cues to scale, depth or anything else. Very little in the images is identifiable or nameable, except the overall concept.

**Figure 6:**
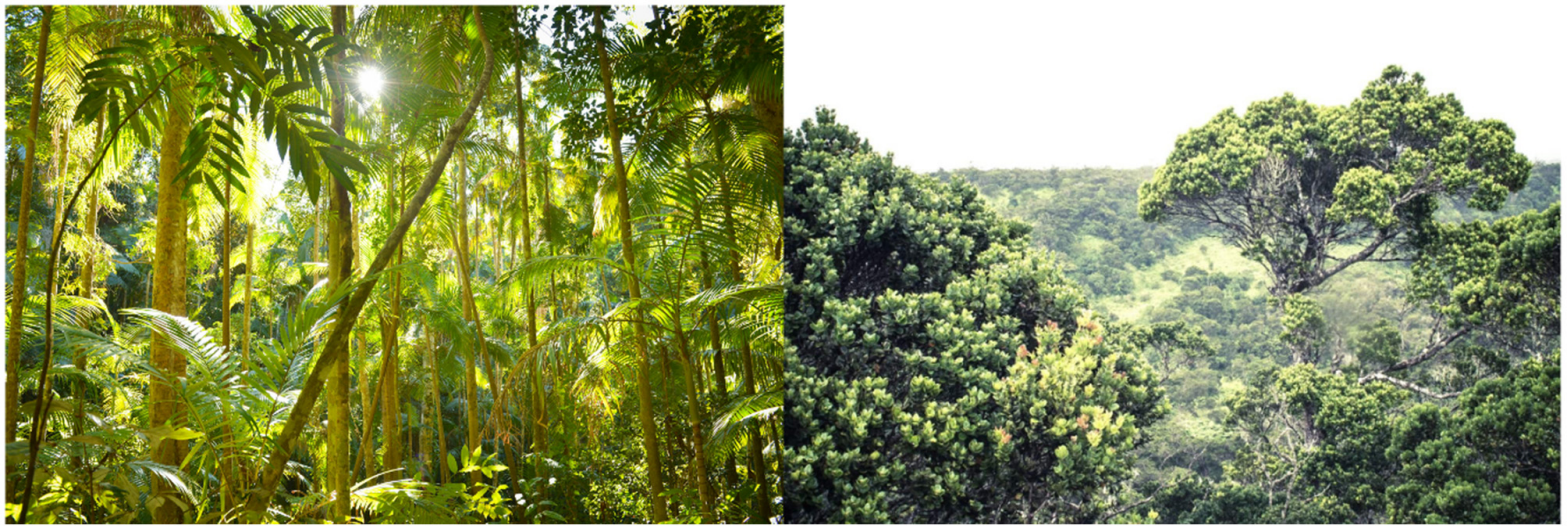
Jungle scenes have intermediate levels of order, Cartesian grids and conceptual density, compared to human environments and inanimate environments. Photo credits left: Rosa Stone, and right: pixnio.com. CC0 licenses.

A variety of things in human environments are not designed by humans, including: people, animals, trees, plants, rocks, the sky, dust and detritus. People, animals, trees and other plants are not designed by humans, but they are shaped by evolution by natural selection, including the shaping of concepts in animals, including humans. For example, as sources of food or threat. Thus, natural environments designed by evolution, such as jungles, have intermediate order and conceptual density (Figure 6).

People are a special case of non-designed things in human environments (seen in Figures 2–4). We are shaped by evolution to read human faces and bodies for emotion and intent, and we are shaped to interact with other humans for survival, reproduction and social benefit. Thus, although people are not reified concepts, they are heavily conceptualized. People are also mostly covered in clothes, designed in part to be easily seen and distinguished from the environment, and to indicate to others whatever they want to indicate, e.g., gender, class, national identity or profession.

Inanimate, non-designed things, such as rocks, the sky, dust and detritus (Figure 5), may be present in human environments, but we generally don't see them, or we ignore them, or we get rid of them. A fair amount of effort goes into removing non-conceptual, inanimate entities from our environment, for example, we dust, clean, tidy and remove rubbish from our homes. And similarly, our streets and cities are cleaned of any non-conceptual entities: rubbish, rubble, and anything non-functional or not-designed is removed. The natural increase in entropy reduces the ordered and functional structures of our homes, work and streets, so constant effort is required to maintain and restore the minimal entropy of these environments, which in turn is required to maintain the non-ambiguous conceptual structures of the human environment.

Although most human environments consist mostly of reified concepts, there are many disordered, ambiguous, or hybrid environments, such as slums, ruins, or contemporary art installations. Garages, attics and rubbish dumps can be cluttered, disorganized and high entropy, partly as a result of removing entropy from other spaces into these environments, where the entropy can be safely hidden away and ignored.

In general, people are averse to or ignore non-conceptual entities or experiences, e.g., dust and detritus. However, people can be attracted to certain types of non-conceptual experiences, such as abstract art or walks in Nature. But abstract art is contained safely within a frame, and is generally viewed for a very short time. Walks in Nature, are generally within relatively structured environments, such as a forest. And we may have been selected by evolution to see such environments as restful and attractive.

Although most objects in human environments are reified concepts, these things themselves consist of non-conceptual matter. The design of reified concepts emphasizes perception of the concept, rather than the matter, but the matter or substance of the world is still there, if you look closely. This distinction is related to that between “things” and “objects” theorized by Heidegger and Brown in Thing theory (Heidegger, 1971; Brown, 2001). Thing theory distinguishes between “objects,” which are generally designed things with a function (corresponding to reified concepts) and “things,” which lack function, design or concept. Objects can transform (in our minds) into things, for example if a glass is shattered into pieces. However, we can think of “things” as the space-time distribution of matter, whereas “objects” are the reified concepts in the scene. Thus, things and objects are different ways of seeing the same scene, but in human environments, we often ignore or do not see the things, because we see straight through to the objects.

Objects designed by humans have functions other than to trigger concepts in people, for example, chairs enable people to sit down comfortably, independent of the ability to induce a chair concept in people (although the concept helps identify, locate, and use the chair). So, it is not my contention that reified concepts only function to induce concepts, but rather that this is one of their important functions. And this function is particularly important for understanding why things look the way they do.

## 6 Digital lives

**Figure 7:**
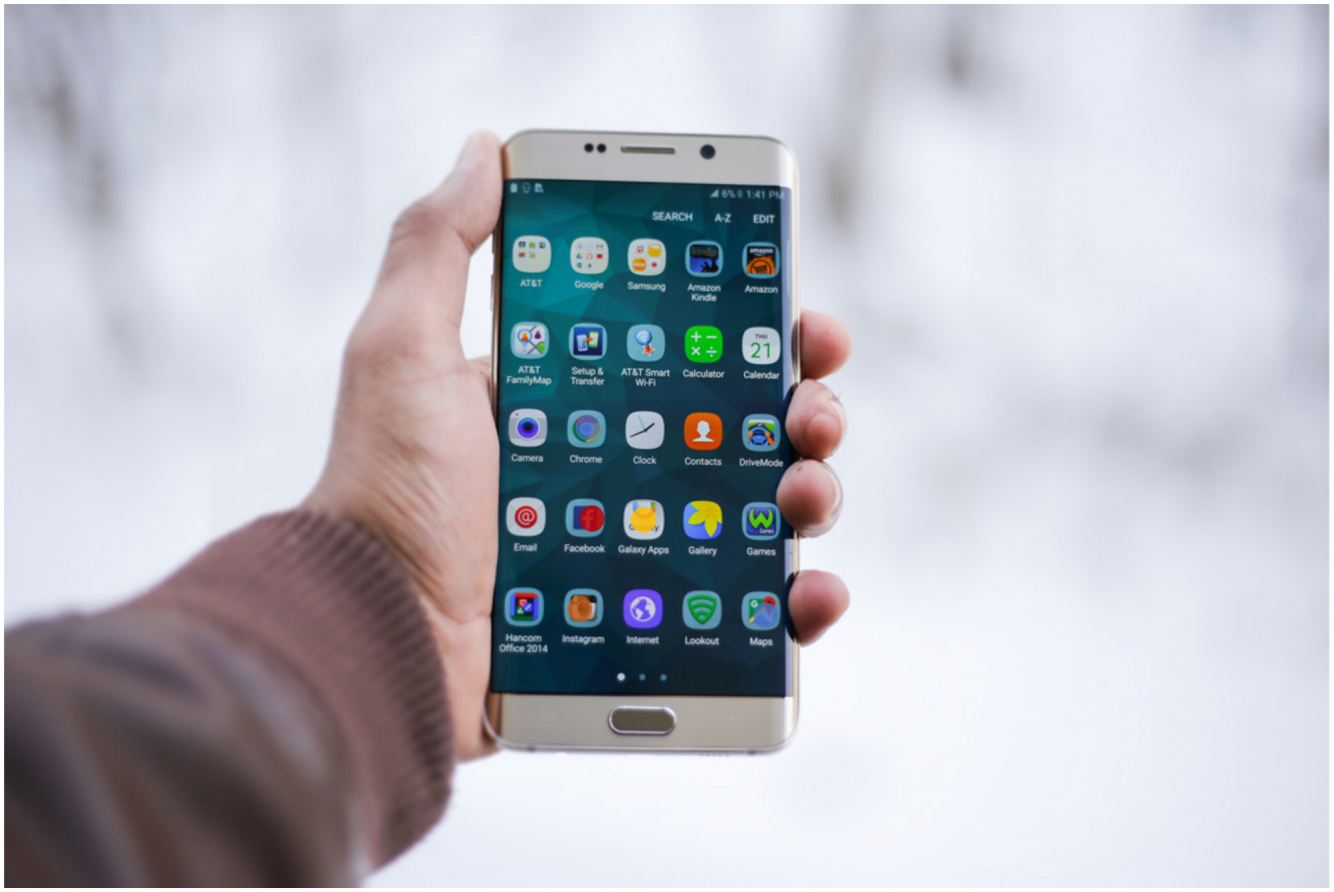
The world of screens and digital content is even more conceptual than other human environments. Note that everything on the screen is reified concepts, in a Cartesian frame, with some nesting, framing and repetition. Photo credit: pxhere.com. CC0 licence.

Modern life is largely lived on screens: phone screens, computer screen and the big screens of TV and films (Figure 7). And the content viewed on these screens is almost entirely conceptual: designed by minds to be consumed by other minds. And the physical substantiation of the concepts on the digital screen is just a means to the end of transfer of concepts from mind to mind. So, the physical screen appears transparent in relation to seeing the concepts themselves. The online world is reduced to pure mind, where the substance of the world seems to have disappeared altogether. Where the screen content represents the physical world, that content is often even more simplified and unambiguous than in the physical world, so that the concepts are conveyed clearly. For example, in a computer game or website, representations of the real world tend to be more iconic of the concept (and lower entropy) than in the real world. And in film, narratives that would play out over days or years in the real world are condensed into minutes, and the narrative determines what is depicted on screen. Thus, the conceptual density and clarity tends to be higher on screen than in the real world.

The spatiality of most screen worlds is more two dimensional than the real world due to being presented on a two-dimensional screen. And most screen worlds, such as websites, make little or no effort to simulate the depth dimension, so that the viewer is not situated within the digital scene. The impression of being within the digital scene is what virtual reality creates, and the absence of this in most screen worlds may contribute to the impression that these worlds are entirely conceptual, rather than being “real” in the sense of being located in space and time.

The modern world of reified concepts may have made it easier for us to get used to the even more conceptual screen world. Or in the other direction, the modern screen world may make it easier for us to see the non-screen human world as composed of reified concepts, where the substance of the world is invisible. The advent of AI, and the embedding of AI in all our devices and machines, further expands the mind into the physical world, and further dissolves the distinction between the physical and the mental, resulting in hybrid mental/physical things, such as cars that drive and converse with you. As AI takes on more mental functions and is embedded into more physical things, these mental functions become reified; so that “extended mind theory” becomes hard to deny (Hutchins, 1995; Clark and Chalmers, 1998).

## 7 One day in Genoa

**Figure 8:**
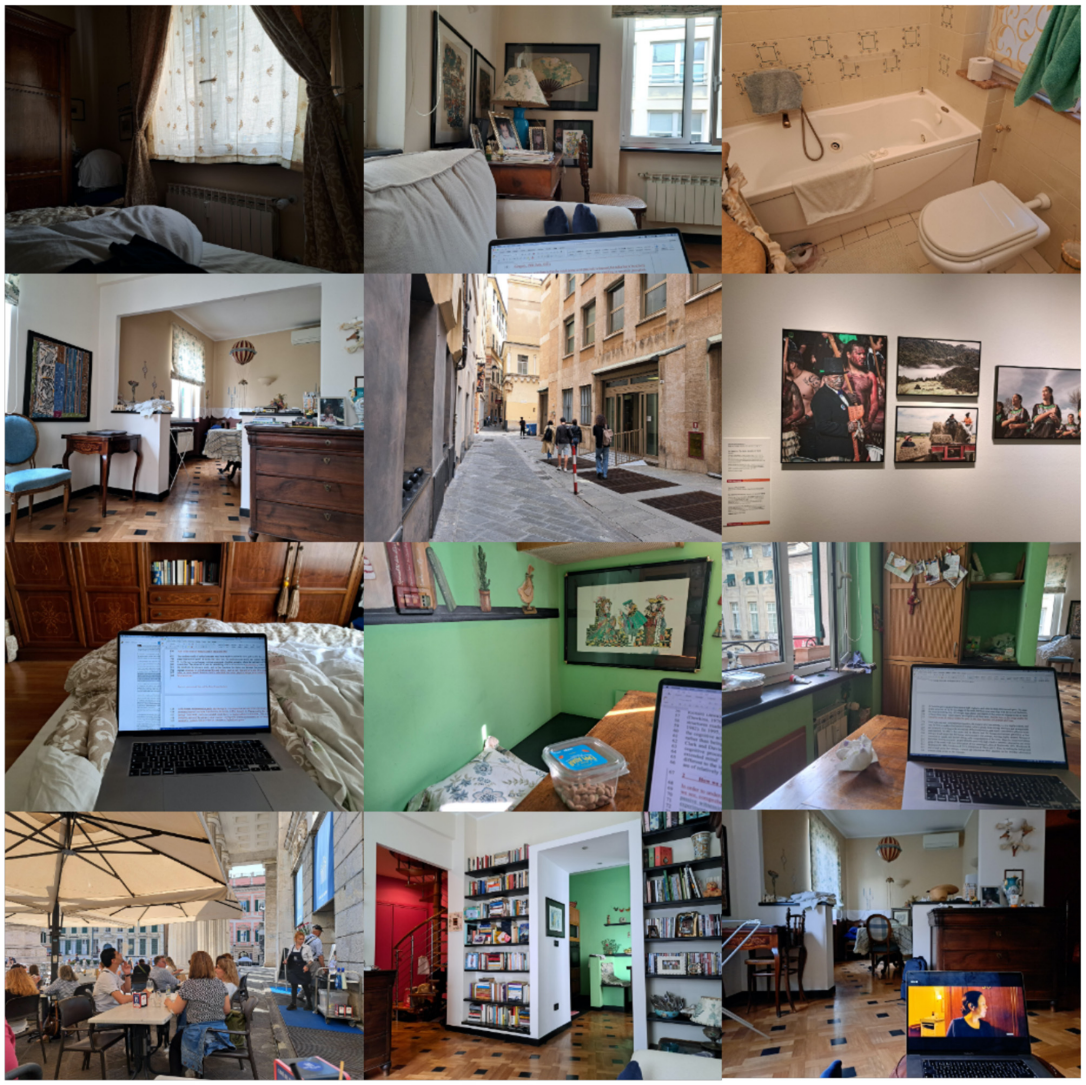
Scenes from my day. I set an hourly alarm for 12 h in Genoa, and when the alarm went off, I took a photograph of whatever was in front of me. Almost everything in these photographs appears to me to be reified concepts, embedded in Cartesian grids.

I have claimed that most human environments consist mainly of reified concepts, orientated to a Cartesian grid, but this claim was based on personal experience and giving some example photographs. In an attempt at a more systematic survey of my environment, I set alarms to go off every hour for 12 h, and then took a photograph of whatever was in front of me when the alarm went off. The results are given in Figure 8. For context: I was on holiday in Genoa in Italy, but I was revising this article according to the reviewers' comments. The photographs confirm that my environments that day consisted mostly of reified concepts, orientated to a Cartesian grid. In fact everything in the photographs appears to be reified concepts. However, clearly this is not a survey of all human environments, and different people may have different ideas about what is seen. So, further work is required to determine the generality of this idea.

## 8 Aesthetics

I argue here that things look the way they do in the human environment, in part, because the human environment consists mainly of reified concepts, arranged within reified conceptual structures, designed to be easily located, perceived and used by our minds. However, it is also true that things look the way they do, in part, because of human aesthetics in design and appreciation. On the other hand, these two explanations may be compatible and complementary. Aesthetics is part of the conceptual design principles used to design our human environments, and what we judge to be aesthetic in these environments may be partly an unconscious judgment of conceptual structure. I am not talking about the aesthetics of high art, but rather the every-day aesthetics of human environments. Domestic environments, such as that of Figure 1, contain only simple, geometric, and symmetric objects, arranged in a geometric pattern, of low entropy, because of every-day aesthetics of tidiness, order and “looking nice.” But these every-day aesthetics may be unconscious judgments of how conceptually comprehensible the scene is.

There is a variety of evidence that everyday aesthetics is based on an unconscious judgment of order/low entropy and/or ease of understanding the scene. In the 1920s and 30s, George Birkhoff argued that the aesthetics (M) of an object or scene can be measured as the ratio of the order (O) to the complexity (C) of the scene: M = O/C (Birkhoff, 1933), i.e., things of high order and low complexity are judged to be aesthetic, e.g., a Palladian villa. In the 1960s and 70s, Max Bense proposed a similar measure, based on information theory and information processing: M = R/H, where R is the redundancy and H the entropy of the object or scene (Bense, 1969). In the 1990s, Jürgen Schmidhuber argued aesthetics can be measured as the shortness of the description of the object or scene, which is again related to the order or comprehensibility of the scene (Schmidhuber, 1997). More recently, Reber, Schwarz, and Winkielman argued that judgments of beauty are determined by the ease with which the information in a scene can be cognitively processed (Reber et al., 2004). Thus, there is a long tradition, and accumulating evidence, that everyday aesthetics is based on an unconscious judgment of order/low entropy, related to the ease of understanding a scene.

However, aesthetics involves factors in addition to cognitive fluency, including emotion, culture and art. Emotion is often part of the aesthetic response, but cognitive fluency (or the lack of it) may evoke that emotion. Culture affects aesthetic response, but this may be partly by affecting cognitive fluency. Art is beyond the scope of this article, but prior to the Renaissance, visual art mainly involved representing (or manifesting) concepts, e.g., in the Lascaux caves or religious icons. But from the Renaissance, western art pursued a realistic pictorial representation of reality. And after the invention of photography, western art moved beyond conceptual or realistic representation, to focus more on subjective experience. Thus, visual art today may involve reified concepts, but it is rarely about representing or manifesting common concepts. However, the appreciation of art also involves an aesthetic response, which may be partly determined by the cognitive fluency of the experience.

## 9 Discussion

To summarize, I argue that: (1) Most human environments now consist mainly of reified concepts. (2) These externalized concepts look like simple cartoons of the concepts they reify, with flat, homogenous surfaces in geometric shapes, with one or a small number of colors, textures and surfaces, so that they can be easily identified and distinguished. (3) These concepts are often organized and structured in the environment by being: orientated to a Cartesian grid, nested within larger concepts and framed to group concepts; and sometimes consisting of multiple near-identical units. (4) Non-conceptual content is actively removed from human environments, resulting in a very low entropy of information, and giving the illusion that reality is conceptual. (5) Most of these features of human environments help perception, comprehension and memory of those environments. (6) Understanding that human environments consist mainly of structured, reified concepts, explains a number of features of how they look.

If the world around us is mostly externalized concepts, within a conceptual structure, then political questions arise: whose concepts and with what purpose and agenda? Do different cultures, genders and power groups have different externalized concepts? Do the externalized concepts impose concepts and power structures upon the viewer? There is no space to address these questions here, but this is an example of how the idea can stimulate further thinking.

It would also be interesting to investigate the relationship between the conceptual content/structure of particular environments and how people feel in those spaces. For example, does the entropy or concept readability of environments affect peoples' stress/anxiety levels? Potential applications in design, architecture and environmental psychology might include explicit consideration of: the reified concepts involved, the readability of those concepts, the entropy and the Cartesian grid.

I have restricted myself to discussing how things look, but these ideas could be extended to how things sound. For example, do we restrict permissible sounds in human environments to meaningful sounds, eliminating non-conceptual sounds as “noise,” and do we seek low entropy sound environments, where meaningful sounds are easily discriminated?

I have restricted myself here mainly to considering static perception and concepts, rather than considering dynamic concepts and how things look over time. For example, we may “see” “the cat sitting down on the mat” i.e., we may “see” changes in the environment in the form of subject, verb and object sentences, corresponding to complex concepts. An important dynamic concept that our minds attribute to a scene changing over time is causation, and the derivative concept of intension. We “see” causation and intension in a changing scene, for example: “The cat ate the mouse.” As Gibson (1979) pointed out with his concept of “affordances,” human environments are designed to enable us to do things, for example, cups are designed to enable us to drink, kitchens are designed to enable us to cook, and streets are designed to enable us to travel. And in some cases, the concept of how to do these things is embedded in the designed object or environment, rather than pre-existing in the user's head (Gibson, 1979), e.g., a cup guides us how to pick it up, and a street guides us through it. Most of the causation we see occurring in human environments was designed by other minds using concepts, which we can then “see” in that environment, or can acquire by using that environment.

Another possible extension of this idea is the externalized mind. Hutchins (1995) and Clark and Chalmers (1998) proposed that objects can be part of a cognitive process and thereby function as extensions of the mind. Externalized concepts may be one aspect of distributed cognition and extended mind, i.e., humans may have externalized various aspects of mind into the environment, including books, machines, the internet, distributed cognition and externalized concepts. And these may work together to determine the behavior of societies, within which peoples' behavior is directed by externalized concepts, such as shops, schools, businesses and governments.

Limitations of the current work include: (1) The very limited set of environments considered, and this needs to be extended in a more systematic way in order to test the generality of the conclusions. (2) The methodology is anecdotal in part, and some of the ideas need empirical testing. (3) The work considers how I see the world, but needs to be extended to how others see the world, for example from neurodiverse or non-Western perspectives. (4) The non-conceptual and non-design aspects of human environments, and how these interact with the conceptual aspects, need to be considered.
